# *BRCA1* and *BRCA2* germline mutations in Chinese Hakka breast cancer patients

**DOI:** 10.1186/s12920-023-01772-9

**Published:** 2024-01-02

**Authors:** Yinmei Zhang, Heming Wu, Caiyan Gan, Hui Rao, Qiuming Wang, Xueming Guo

**Affiliations:** 1 Center for Precision Medicine, Meizhou People’s Hospital (Huangtang Hospital), Meizhou Academy of Medical Sciences, No 63 Huangtang Road, Meijiang District, Meizhou, 514031 P. R. China; 2 Guangdong Engineering Technological Research Center of Clinical Molecular Diagnosis and Antibody Drugs, Meizhou, China; 3 Department of Medical Oncology, Meizhou People’s Hospital (Huangtang Hospital), Meizhou Academy of Medical Sciences, Meizhou, People’s Republic of China

**Keywords:** Breast cancer, *BRCA1* mutation, *BRCA2* mutation, Next generation sequencing

## Abstract

**Objective:**

To investigate the prevalence of *BRCA1/2* gene variants and evaluate the clinical and pathological characteristics associated with these variants in Chinese Hakka breast cancer patients.

**Methods:**

A total of 409 breast cancer patients were analyzed based on next-generation sequencing results, with 337 categorized as non-carriers and 72 as carriers of *BRCA1/2* variants. Data on the patients’ *BRCA1/2* gene mutation status, clinical and pathological characteristics, as well as menstrual and reproductive information, were collected, analyzed, compared, and tabulated. Logistic regression analysis was performed to explore the relationship between clinical characteristics and pathogenic variants.

**Results:**

Among the patients, 72 were identified as carriers of pathogenic or likely pathogenic variants in *BRCA1/2*, while 337 had likely benign or benign mutations. The *BRCA1* c.2635G > T (p. Glu879*) variant was detected at a high frequency, accounting for 12.5% (4/32) of the *BRCA1* mutations, while the c.5164_5165del (p.Ser1722Tyrfs*4) variant was common among the *BRCA2* mutations, accounting for 17.5% (7/40). It was observed that a higher proportion of *BRCA1* carriers had the triple-negative breast cancer subtype, whereas more *BRCA2* carriers exhibited estrogen receptor (ER) + and progesterone receptor (PR) + subtypes. Multivariate logistic regression analysis revealed that a family history of cancer (OR = 2.36, 95% CI = 1.00–5.54), bilateral cancer (OR = 4.78, 95% CI 1.61–14.20), human epidermal growth factor receptor 2 (HER2)- (OR = 8.23, 95% CI 3.25–20.84), and Ki67 ≥ 15% (OR = 3.88, 95% CI 1.41–10.65) were associated with *BRCA1/2* mutations, with the age at diagnosis, age at menarche, and premenopausal status serving as covariates.

**Conclusions:**

The most common pathogenic variant of the *BRCA1* and *BRCA2* in breast cancer patients was c.2635G > T and c.5164_5165del, respectively. Additionally, a family history of cancer, bilateral cancer, HER2-, and Ki67 ≥ 15% were identified as independent predictors of *BRCA1/2* pathogenic variants.

**Supplementary Information:**

The online version contains supplementary material available at 10.1186/s12920-023-01772-9.

## Background

Female breast cancer is a widespread and significant health concern, ranking as the fifth leading cause of cancer-related deaths [1]. Understanding the various factors contributing to the development of breast cancer in women is crucial, as it involves a complex interplay of genetic, reproductive, lifestyle, and environmental influences [2]. One particularly noteworthy factor in hereditary breast cancer is the presence of pathogenic variants in the breast cancer susceptibility genes *BRCA1* and *BRCA2* [3]. These genes play a crucial role in DNA damage repair and act as tumor suppressors, contributing to genome stability [4]. Carriers of *BRCA1* and *BRCA2* mutations face a cumulative breast cancer risk of 72% and 69% respectively by the age of 80 [5]. Consequently, germline genetic screening for *BRCA1/2* mutations has become an essential tool for cancer prediction and clinical management, enabling carriers to benefit from surveillance, chemoprevention, and preventive surgery to mitigate breast cancer risk [6]. Moreover, individuals with metastatic breast cancer and *BRCA1/2* mutations can benefit from treatment with poly ADP-ribose polymerase inhibitors [7] or in combination with cisplatin [8]. Hence, genetic counseling and testing are imperative in the context of hereditary breast cancer.

A family history of cancer, negative human epidermal growth factor receptor 2 (HER2), high Ki67 index, and lymph node status have been identified as closely associated with *BRCA* mutations [9]. Additionally, Khalis et al. [10] observed a significant association between menstrual history, fertility status, and an increased risk of breast cancer. Indeed, this article explores the potential influence of the *BRCA1/2* gene on clinical and pathological features, as well as on ovulation and the menstrual cycle.

Hakka is a Han nationality group with a unique genetic background. Hakka mainly lives in southern China and has a wide distribution in Southeast Asia [11]. However, there is limited reporting on the *BRCA1/2* mutation sites and their associated clinical and pathological characteristics, as well as menstrual and reproductive status among the Chinese Hakka population. Consequently, this article aims to investigate the clinical and pathological features, menstrual patterns, and reproductive conditions in individuals carrying germline *BRCA1/2*.

## Materials and methods

### Participants

This retrospective study included 409 breast cancer patients who were admitted to Meizhou People’s Hospital (Huangtang Hospital), Meizhou Academy of Medical Sciences, between September 2017 and November 2021. The inclusion criteria were as follows: (1) female patients who had been diagnosed with breast cancer; (2) undergoing *BRCA* gene testing; and (3) complete clinical records, including clinical characteristics and menstrual and reproductive case data. Exclusion criteria involved cases where the genetic test results were of uncertain significance. This study was approved by the Ethics Committee of Medicine, Meizhou People’s Hospital (Huangtang Hospital), Meizhou Academy of Medical Sciences. All participants signed informed consent by the Declaration of Helsinki.

### *BRCA1/2* testing

Approximately 2 mL of peripheral blood was collected in a tube containing EDTA, and genomic DNA was extracted according to the QIAamp DNA Blood Mini Kit instructions (Qiagen, Germany). The genomic DNA samples were sent to CapitalBio (Beijing, China) and subjected to next-generation sequencing on the Ion Proton instrument (Life Technologies). All procedures were performed according to the standard operating procedures of the Life Technology Company. The sequencing results were compared with the *BRCA1* (NM_007294.3) and *BRCA2* (NM_000059.3) reference sequences for variant detection. According to the Human Genome Variation Society (HGVS) guidelines, there are five grades of variants: pathogenic variants, likely pathogenic variants, variants of uncertain significance (VUS), likely benign variants, and benign variants [12].

This study divided breast cancer patients who had been tested for *BRCA1/2* gene variants into two groups: *BRCA1/2* mutation carriers with pathogenic and likely pathogenic variants, and non-*BRCA1/2* mutation carriers. The demographic data, clinical and pathologic characteristics, and menstrual and reproductive status of the two groups of patients were tabulated, and the two groups of patients were compared.

### Immunohistochemical examination

All of the patients involved in the analysis underwent definitive surgery. The tumor histopathology molecular subtypes were determined by detecting estrogen receptor (ER) and pregnancy receptor (PR) status, HER2 status, and Ki67 marker index. The tumor was defined as ER and/or PR positive if more than 1% of the tumor cells have ER and/or PR positive nuclei. HER2 staining patterns were divided into 4 groups: 3 + (strong and diffuse staining in 10% of cancer cells), 2 + (moderate and diffuse staining), 1 + (local staining), and 0. HER2 positivity was defined as HER2 staining of 3 + and 2 + supplemented with a positive FISH test. HER2- is defined as HER2 staining 0, 1 + , and FISH negative when HER2 staining 2 + . The Ki67 labeling index is expressed as the percentage of positive cells in each case, and the threshold of 15% indicates a high proliferation index.

### Guidance for patient treatment

Breast cancer patients with a diagnosis age younger than 40 years, family history of breast, ovarian, or colorectal cancer; premenopausal breast cancer, bilateral breast cancer; triple-negative breast cancer; HER2-, and Ki67 ≥ 15%, should receive *BRCA* genetic counseling for *BRCA* testing.

Patients with negative genetic test results are considered non-mutant and are advised to undergo regular follow-ups. However, for patients with likely pathogenic or pathogenic mutations, it is essential to explain the risk of carrying the mutated gene to their family members and the possibility of passing it on to their offspring. Moreover, it is recommended to conduct *BRCA1/2* genetic testing for immediate family members of these patients.

For women with a confirmed pathogenic or likely pathogenic variant of *BRCA1/2* or those with a high degree of suspicion based on the presence of a known or possibly pathogenic variant in the family, post-test counseling should encompass a discussion of risk-reducing mastectomy. This counseling should include an exploration of the degree of cancer risk reduction/protection, surgery-related risks, breast reconstruction options, management of menopausal symptoms, and discussions about reproductive requirements [13]. Additionally, Olaparib and other PARP inhibitors can be used as maintenance therapy in breast cancer patients with *BRCA1/2* mutations [14]. Carboplatin may be recommended in patients with advanced triple-negative breast cancer with *BRCA1/2* mutations.

### Statistical analysis

SPSS statistical software version 22.0 was used for data analysis. Means ± SDs were used to evaluate differences in quantitative data and N (%) described qualitative data. Student’s t-test was used to compare continuous variables, and the χ^2^ test was used to compare categorical variables. Log-rank test was used to compare the age of breast cancer onset in different mutation states. Odds ratios (ORs) and 95% confidence intervals were calculated by logistic regression. All *p* values were two-sided, and *p* < 0.05 was considered statistically significant.

## Results

### Pathogenic and likely pathogenic *BRCA1/2* mutations in the Hakka breast cancer patients

**Fig. 1:**
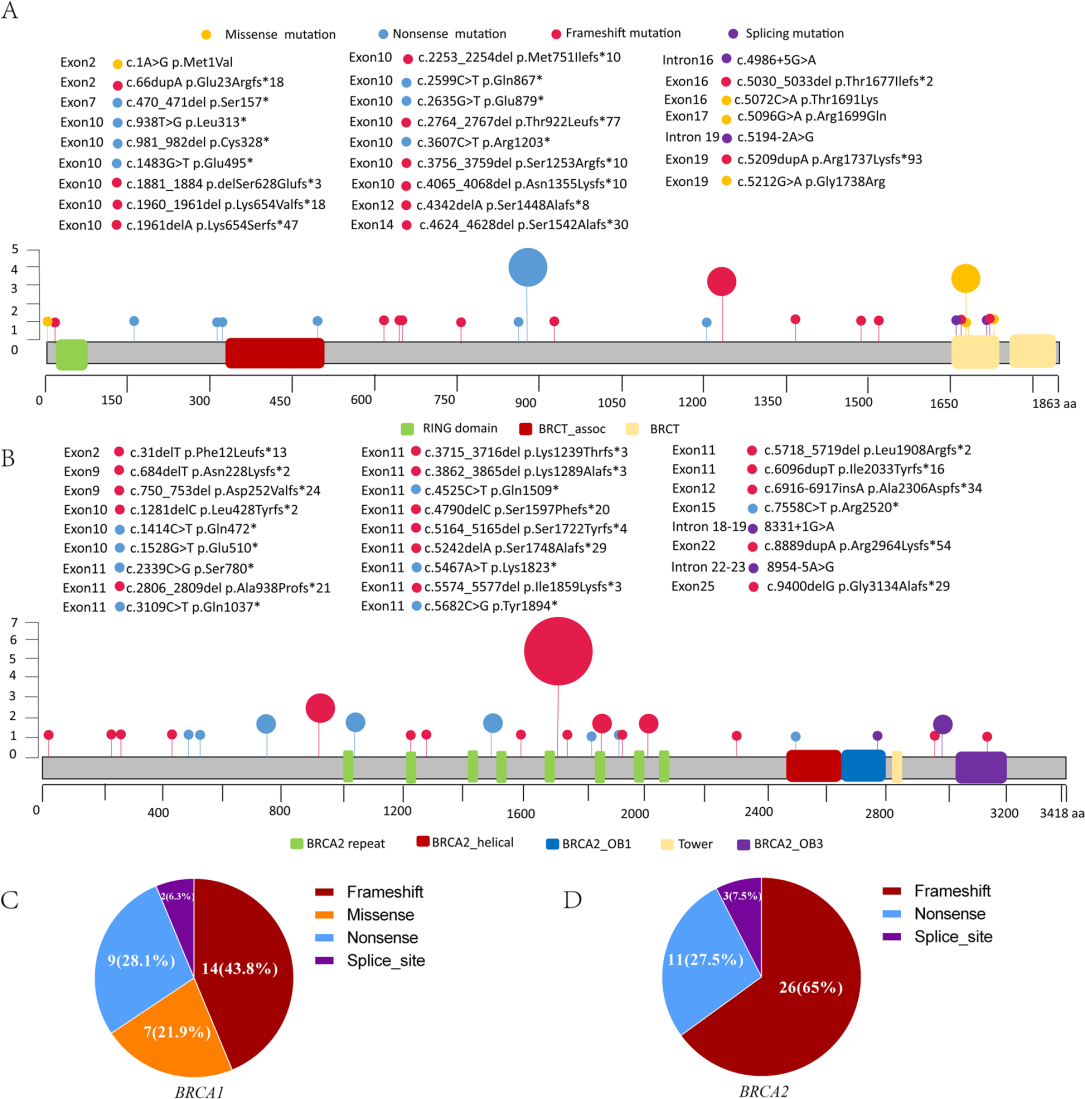
Locations and frequency of mutation sites in *BRCA1* and *BRCA2*. (**A**) Twenty-five mutation sites in the *BRCA1* gene (**B**) Twenty-six mutation sites in the *BRCA2* gene. **C** The number and proportion of different variant types in the *BRCA1* gene. **D** The number and proportion of different variant types in the *BRCA2* gene

In this study, Twenty-five different *BRCA1* mutations were identified, comprising twenty-two pathogenic variants and three likely pathogenic variants. The most frequently observed *BRCA1* was c.2635G > T (p. Glu879*), accounting for 12.5% (4/32) of the *BRCA1* mutations (Fig. 1A, Table S1). Among these variants, twenty-three variants were located in exons (exons 2, 7, 10, 12, 14, 16, 17, and 19), and two were located in introns, with the most frequently mutated exon being exon 10, observed in 18 breast cancer patients (Fig. 1A). The predominant mutation type was frameshift mutation (14/32, 43.8%), followed by nonsense mutations (10/32, 31.3%), missense mutations (6/32, 18.8%), and intron mutations (2/32, 6.3%) (Fig. 1C). Frameshift mutation and nonsense mutation at any location of *BRCA1* will make the *BRCA1* gene unable to correctly encode the *BRCA1* protein. Pathogenic missense mutations and splicing mutations, occurring in conserved regions, may impact protein structure and function, except for *BRCA1* c.1A > G, which occurs at the start codon, rendering the *BRCA1* protein untranslatable.

Twenty-six different *BRCA2* mutations were identified in breast cancer patients, comprising twenty-five pathogenic variants and a likely pathogenic variant (Table S1). The most frequent *BRCA2* variant was c.5164_5165del (p. Ser1722Tyrfs*4), accounting for 17.5% (7/40) of the mutations (Fig. 1B, Table S1). Notably, a frameshift mutation *BRCA2* c.6916-6917insA (Ala2306Aspfs*34), was identified, which has not been previously reported or listed in the ClinVar database and the *BRCA* Exchange database. Among the identified variants, twenty-four variants were in exons (2, 9, 10, 11, 12, 15, 22, and 25), and two were in introns. The most frequently mutated exon was exon 11, detected in 27 breast cancer patients (Fig. 1B). The predominant mutation type among the *BRCA2* mutations was frameshift mutation (26/40, 65%), followed by a nonsense mutation (11/40, 27.5%) and an intron mutation (3/40, 7.5%) (Fig. 1D). It is important to note that frameshift and nonsense mutations at any location of *BRCA2* can result in the inability to correctly encode the *BRCA2* protein. Additionally, splicing mutations occur in the C-terminus, which binds to DNA.

### Clinical pathologic characteristics

**Fig. 2:**
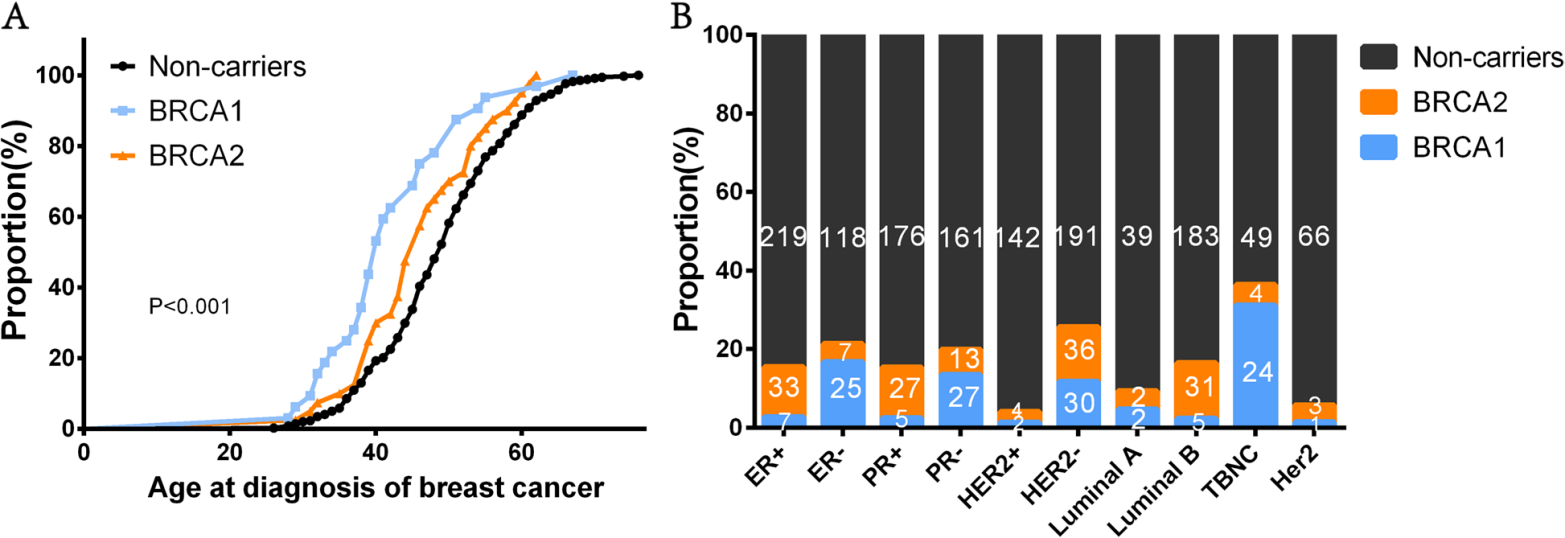
Age at onset of breast cancer and clinicopathologic characteristics of *BRCA1/2* mutation carriers and non-carriers. **A** Age at onset of breast cancer by mutation status. **B** Clinicopathologic characteristics of *BRCA1* mutation carriers, *BRCA2* mutation carriers, and non-carriers

The clinical characteristics of the study patients are presented in Table 1. The mean age at diagnosis for *BRCA1/2* mutation carriers was 43.78 ± 9.06 years, ranging from 28 to 67 years, which was 4.66 years earlier than non-carriers (48.44 ± 9.20, ranging from 26 to 76 years) (*P* < 0.001). Notably, there was a significant difference in the age of breast cancer onset under different mutation states (*P* < 0.001) (Fig. 2A). *BRCA1* mutation carriers were diagnosed at a younger age, with 53% (17/32) of *BRCA1* carriers diagnosed before the age of 40 years. Among 409 breast patients, 35 had a family history of cancer, and the proportion of *BRCA1/2* mutation carriers (13/72, 18.1%) was higher than that of non-carriers (22/337, 6.5%), representing a significant difference (*P* = 0.002). *BRCA1/2* mutation carriers had a higher rate of bilateral breast cancer (9/72, 12.5%) compared to non-carriers (11/337, 3.3%). This difference was statistically significant (*P* = 0.008). Over 95% of the patients had invasive breast cancer, with no significant difference between *BRCA1/2* mutation carriers and non-carriers in the rate of invasive breast cancer (Table 1).

**Table 1:** Clinical characteristics in g*BRCA1/2* carriers and noncarriers

Categories	Total	Noncarriers	*BRCA1*/2 mutation carriers	*P* value
Number of patients	409 (100%)	337 (82%)	72 (18%)	
Mean age at diagnosis (years)	47.62 ± 9.34	48.44 ± 9.20	43.78 ± 9.06	< 0.001
Range	26–76	26–76	28–67	
< 40 years	91 (22.2%)	64 (19%)	27 (37.5%)	0.001
≥ 40 years	318 (77.8%)	273 (81%)	45 (62.5%)	
With family history of any cancer				0.002
No	374 (91.4%)	315 (93.5%)	59 (81.9%)	
Yes	35 (8.6%)	22 (6.5%)	13 (18.1%)	
Laterality breast cancer				
Bilateral	20 (4.9%)	11 (3.3%)	9 (12.5%)	0.008
Right	189 (46.2%)	162 (48.1%)	27 (37.5%)	
Left	200 (48.9%)	164 (48.7%)	36 (50%)	
Invasive carcinoma	389 (95.1%)	320 (95%)	69 (95.8%)	0.99
None invasive	20 (4.9%)	17 (5%)	3 (4.2%)	
AJCC stage				0.635
0–1	61 (14.9%)	52 (15.4%)	9 (12.5%)	
2	195 (47.7%)	160 (47.5%)	35 (48.6%)	
3	121 (29.6%)	101 (30%)	20 (27.8%)	
4	32 (7.8%)	24 (7.1%)	8 (11.1%)	
T				0.857
0–1	115 (31.1%)	95(28.2%)	20(27.8%)	
2	229 (56%)	190(56.4%)	39(54.2%)	
3	31 (7.6%)	26(7.7%)	5(6.9%)	
4	25 (6.1%)	20(5.9%)	5(6.9%)	
Unknown	9 (2.2%)	6(1.8%)	3(4.2%)	
N				0.397
0	166(40.6%)	140(41.5%)	26(36.1%)	
1	110(26.9%)	89(26.4%)	21(29.2%)	
2	64(15.6%)	52(15.4%)	12(16.7%)	
3	63(15.4%)	53(15.7%)	10(13.9%)	
Unknown	6(1.5%)	3(0.9%)	3(4.2%)	
M				0.253
0	377 (92.2%)	313 (92.9%)	64 (88.9%)	
1	32 (7.8%)	24 (7.1%)	8 (11.1%)	
ER status				0.132
Positive	259 (63.3%)	219 (65%)	40 (55.6%)	
Negative	150 (36.7%)	118 (35%)	32 (44.4%)	
PR status				0.231
Positive	208(50.9%)	176 (52.2%)	32 (44.4%)	
Negative	201(49.1%)	161 (47.8%)	40 (55.6%)	
HER2 status				< 0.001
Positive	148(36.2%)	142 (42.1%)	6 (8.3%)	
Negative	257(62.8%)	191 (56.7%)	66 (91.7%)	
Unknown	4(1.0%)	4 (1.2%)	0 (0%)	
Ki67 status				0.010
≥ 15%	338 (82.6%)	271 (80.4%)	67 (93.1%)	
< 15%	71 (17.4%)	66 (19.6%)	5 (6.9%)	
Molecular subtype				< 0.001
Luminal A	43 (10.5%)	39 (11.6%)	4 (5.6%)	
Luminal B	219 (53.5%)	183 (54.3%)	36 (50%)	
TNBC	77 (18.8%)	49 (14.5%)	28 (38.9%)	
HER2	70 (17.1%)	66 (19.6%)	4 (5.6%)

The distribution of *BRCA1/2* mutation carriers in 0–1, 2, 3, and 4 stages was 9 (12.5%), 35(48.6%), 20 (27.8%), and 8 (11.1%), respectively, with no significant difference observed between *BRCA1/2* mutation carriers and non-carriers (52, 15.4%; 160, 47.5%; 101, 30% and 24, 7.1%). Similarly, there were no significant differences in the numbers of breast cancer patients in the T, N, and M stages in *BRCA1/2* mutation carriers and non-carriers.

The data reveals that 25(78.1%) *BRCA1* carriers had ER-, while more non-carriers and *BRCA2* carriers had ER + . The expression of ER in *BRCA1* carriers significantly differed from non-carriers (*P* < 0.001) and *BRCA2* carriers (*P* < 0.001). Additionally, 27(84.4%) *BRCA1* carriers had PR-, compared to 161(47.8%) non-carriers and 13(32.5%) *BRCA2* carriers with PR-. The expression of PR in *BRCA1* carriers was significantly different from non-carriers (*P* < 0.001) and *BRCA2* carriers (*P* < 0.001). The most common molecular subtype was TNBC in *BRCA1* carriers (24, 75%), whereas the most common molecular subtype was Luminal B in *BRCA2* carriers (31, 77.5%) and non-carriers (183, 54.3%) (Fig. 2B).

Furthermore, the number of *BRCA1/2* mutation patients with pathological results of ER-, PR-, HER2-, Ki67 ≥ 15% were 32 (44.4%), 40 (55.6%), 66 (91.7%), and 67 (93.1%), respectively. In comparison, the number of patients without *BRCA1/2* mutations with ER-, PR-, HER2-, and Ki67 ≥ 15% were 118 (35%), 161(47.8%), 191 (56.7%), 271(80.4%), respectively. In conclusion, *BRCA1/2*-mutated breast cancers were likely to be HER2- (*P* < 0.001) and Ki67 ≥ 15% (*P* = 0.010).

### Menstrual and reproductive status of breast cancer patients

The average age at menarche in individuals without the *BRCA1/2* mutation was 15.61 ± 1.84 years, whereas in patients with the *BRCA1/2* mutation, it was slightly younger at 15.14 ± 1.80 years. The difference in age at menarche between the two groups was statistically significant (*P* = 0.047). Additionally, 18.1% of breast cancer patients with *BRCA1/2* variants had menarche ages younger than 13 years, compared to only 12.2% of breast cancer patients with non-*BRCA* variants. Among *BRCA1/2* mutation carriers, 55 (76.4%) of breast cancer patients experienced the onset of breast cancer before menopause, while 17 (23.6%) experienced it after menopause. This was significantly different from non-carriers, with more individuals developing breast cancer before menopause among *BRCA*-mutated patients (*P* = 0.026). The mean age of first breastfeeding and natural menopause was 23.88 ± 3.77, and 50.52 ± 3.83 in non-carriers, respectively, compared with 23.38 ± 2.87 years, and 49.60 ± 3.44 years in *BRCA1/2*-mutated patients. The mean age at first lactation and natural menopause did not show significant differences between *BRCA1/2*-mutated patients and non-carriers. Furthermore, the number of *BRCA1/2*-mutated patients with menstrual periods duration less than 3, 4 to 7, or more than 8 days was 5 (6.9%), 65(90.3%), 2 (2.8%) respectively, and the number with menstrual cycles less than 25, 25 to 35, more than 35 days were 3 (4.2%), 66 (91.7%), 3 (4.2%), respectively. χ^2^ statistics showed that menstrual period duration and menstrual cycle were similar to those of non-carriers.

The average number of children born to *BRCA1/2*-mutated patients was 2.15 ± 1.13 babies, which was similar to non-*BRCA* mutation patients (2.20 ± 1.05 babies). The duration of breastfeeding was 21.07 ± 13.47 months, and the average breastfeeding duration per child was 9.34 ± 4.69 months in non-carriers, while the duration of breastfeeding was 17.07 ± 9.84 months, and the average child breastfeeding duration was 8.10 ± 2.31 months in *BRCA1/2*-mutated patients. Breastfeeding time in *BRCA1/2* mutation carriers was shorter than that of non-carriers, but there was no significant difference. The average number of aborted fetuses was 1.53 ± 0.51 in *BRCA1/2*-mutated patients, and the average number of aborted fetuses was 1.72 ± 1.04 in non-carriers. There were no significant differences in abortion history, the number of abortions, and the rate of recurrent (two or more) abortions between *BRCA1/2*-mutated patients and non-carriers (Table 2).

**Table 2:** Menstrual and reproductive status of *BRCA1*/2 mutation carriers and noncarriers

Categories	Total	Non-carriers	*BRCA1*/2 mutation carriers	*P* value
Number of patients	409 (100%)	337 (82%)	72 (18%)	
Age at menarche (years)	15.53 ± 1.84	15.61 ± 1.84	15.14 ± 1.80	0.047
≤ 13	54 (13.2%)	41 (12.2%)	13 (18.1%)	0.049
14–17	302 (73.8%)	247 (73.3%)	55 (76.4%)	
≥ 18	53 (13.0%)	49 (14.5%)	4 (5.6%)	
Menopausal status				0.026
Premenopausal	266 (65.0%)	211 (62.6%)	55 (76.4%)	
Postmenopausal	143 (35.0%)	126 (37.4%)	17 (23.6%)	
Age at natural menopause (years)	50.42 ± 3.78	50.52 ± 3.83	49.60 ± 3.44	0.374
≥ 45	129 (31.5)	115 (34.1%)	14 (19.4%)	1.000
< 45	8 (2.0%)	7 (2.1%)	1 (1.4%)	
Unknown	272 (66.5%)	215 (63.8%)	57 (79.2%)	
Interval between menarche and menopausal(years)	34.07 ± 4.02	34.12 ± 4.04	33.60 ± 3.94	0.636
Menstrual duration (days)				0.900
≤ 3	33 (8.0%)	28 (8.3%)	5 (6.9%)	
4–7	363 (88.8%)	298 (88.4%)	65 (90.3%)	
≥ 8	13 (3.2%)	11 (3.3%)	2 (2.8%)	
Menstrual cycle (days)				0.714
< 25	15 (3.7%)	12 (3.6%)	3 (4.2%)	
25–35	369 (90.2%)	303 (89.9%)	66 (91.7%)	
> 35	25 (6.1%)	22 (6.5%)	3 (4.2%)	
Reproductive history				0.766
No	12 (2.9%)	9 (2.7%)	3 (4.2%)	
Yes	397 (97.1%)	328 (97.3%)	69 (95.8%)	
Parity	2.19 ± 1.06	2.20 ± 1.05	2.15 ± 1.13	0.729
0	12 (2.9%)	9 (2.7%)	3 (4.2%)	0.678
1–2	260 (63.6%)	221 (65.6%)	39 (54.2%)	
≥ 3	127 (31.1%)	107 (31.8%)	20 (27.8%)	
Unknown	10 (2.4%)	0 (0%)	10 (13.9%)	
Age at first breast-feeding (years)	23.83 ± 3.68	23.88 ± 3.77	23.38 ± 2.87	0.456
< 20	20 (4.9%)	18 (5.3%)	2 (2.8%)	1.000
≥ 20	315 (77.0%)	283 (84.0%)	32 (44.4%)	
Unknown	74 (18.1%)	36 (10.7%)	38 (52.8%)	
The interval between menarche and first breastfeeding (years)	8.31 ± 3.84	8.31 ± 3.88	8.26 ± 3.51	0.948
Duration of breastfeeding(months)	20.71 ± 13.22	21.07 ± 13.47	17.07 ± 9.84	0.113
Every child breast-feeding (months)	9.23 ± 4.54	9.34 ± 4.69	8.10 ± 2.31	0.153
0	4 (1.0%)	4 (1.2%)	0 (0%)	0.31
1–12	312(76.3%)	282 (83.7%)	30 (41.7%)	
13–24	15 (3.7%)	15 (4.5%)	0 (0.0%)	
≥ 25	4 (1.0%)	4 (1.2%)	0 (0.0%)	
Unknown	74 (18.1%)	32 (9.5%)	42 (58.3%)	
Abortion				0.256
Never	259 (63.3%)	216 (64.1%)	43 (59.7%)	
Ever	138 (33.7%)	121 (35.9%)	17 (23.6%)	
Unknown	12 (2.9%)	0 (0%)	12 (16.7%)	
Number of abortions				
1	75 (18.3%)	67 (19.9%)	8 (11.1%)	0.519
2 +	63 (15.4%)	54 (16%)	9 (12.5%)	
Average abortions	1.70 ± 0.99	1.72 ± 1.04	1.53 ± 0.51	0.46

### Logistic regression analysis of the relationship between clinical features and *BRCA1/2* mutation

**Fig. 3:**
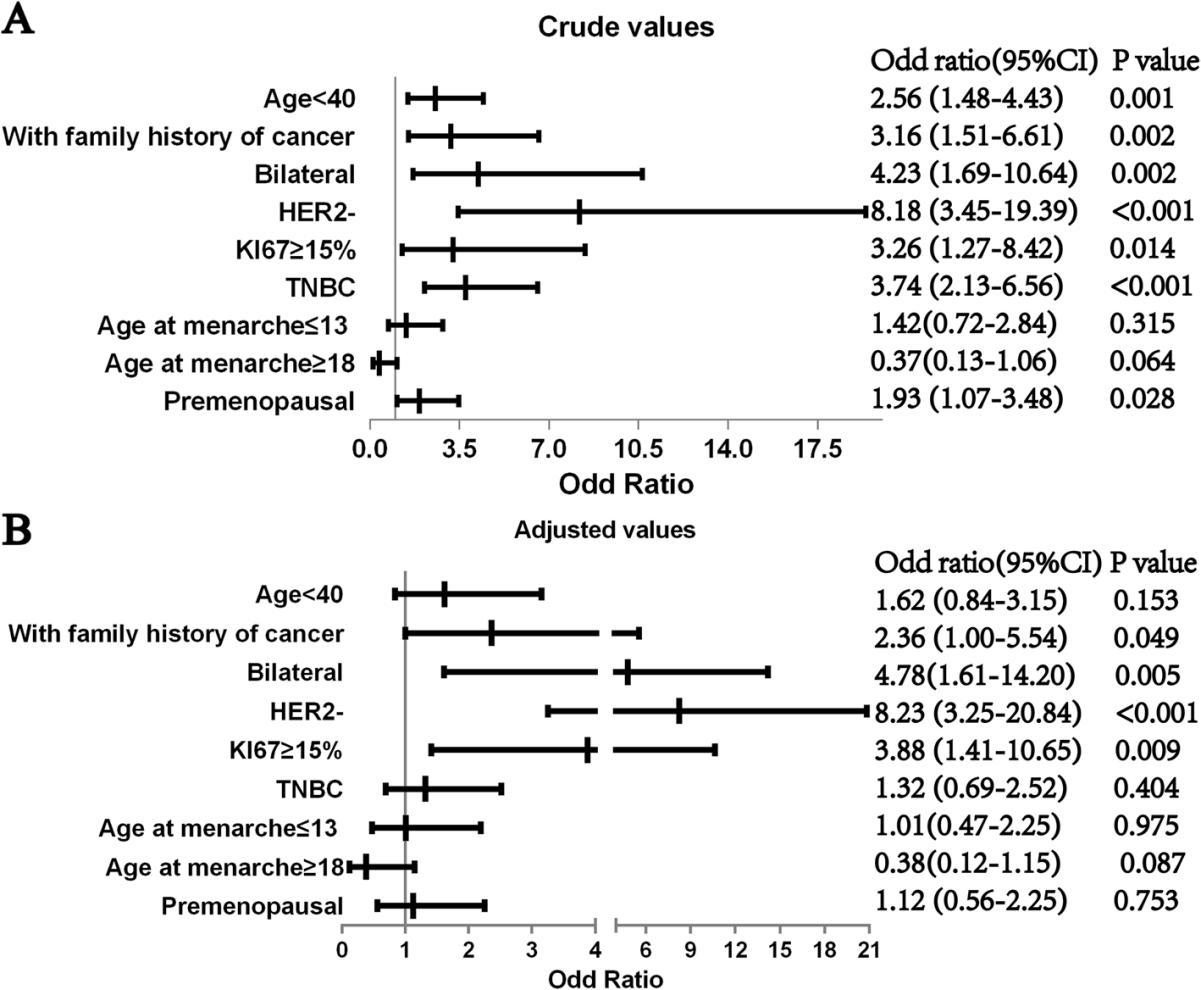
Logistic regression analysis of factors that influence *BRCA1/2* carriers. **A** Univariate logistic regression. **B** Multivariate logistic regression

Logistic regression analysis was performed to identify the clinical features associated with *BRCA* mutation. Univariate logistic regression revealed that being diagnosed at an age younger than 40 years, having a family history, premenopausal breast cancer, bilateral cancer, HER2-, Ki67 ≥ 15% were related to *BRCA* mutation (Fig. 3A). However, multivariate logistic regression demonstrated that having family members with cancer (OR = 2.36, 95% CI = 1.00–5.54), bilateral cancer (OR = 4.78, 95% CI = 1.61–14.20), HER2-(OR = 8.23, 95% CI = 3.25–20.84), Ki67 ≥ 15% (OR = 3.88, 95% CI = 1.41–10.65) were associated with *BRCA1/2* mutation (Fig. 3B).

## Discussion

Breast cancer is the most common cancer and the leading cause of cancer death in women. There are multiple nongenetic and genetic factors for breast cancer. Age, race, early menarche/late menopause, breast characteristics, etc., are the nongenetic factors.

Approximately 5% of breast cancers occur in women under 40 years of age, with the majority being diagnosed in women aged over 50 years [15]. The data from this study reveals that individuals carrying *BRCA1*/2 mutations tend to be diagnosed with breast cancer at a younger age compared to those without the mutations. This trend is particularly noticeable among *BRCA1* mutation carriers, which aligns with findings from previous studies [16, 17]. Early menarche and late menopause have been associated with an increased risk of breast cancer [15]. The pregnancy cycle also influences breast cancer risk due to its direct effects on the metabolism, gene expression profiles, and proliferation dynamics of mammary epithelial cells in response to hormones [18]. Breast characteristics, including proliferative lesions with atypia and dense breast tissue, are associated with increased risk [19, 20]. Family history, *BRCA* mutations, etc., are the genetic factors. *BRCA1* and *BRCA2* play an important role in maintaining genome stability by promoting efficient and accurate repair of double-strand breaks [21]. It has been reported that 5–10% of breast cancer cases result from germline mutations of *BRCA* genes [22]. For breast cancer, initially, the primary tumor, lymph node, and metastasis (TNM) are a standardized classification system for making integrated judgments and precise decisions [23, 24]. With the rapid development of knowledge of cancer biology and the discovery and validation of biological factors, ER, PR, HER2, histological grade, and multigene prognostic assays, into the staging system [25]. Ki67 is a well-known proliferative marker for evaluating cell proliferation. It is highly expressed in malignant cells but almost undetectable in normal cells, and the Ki67 index independently predicts cancer progression [26]. Van der Groep et al*.* reported that in the age group under 54 years, the likelihood of *BRCA1*-related disease was only 9% when Ki67 was low, but as high as 75% when Ki67 was high [27].

The *BRCA* gene mutation sites are associated with different populations. In the Chinese Hakka population, the most frequent mutation in the *BRCA1* gene was c.2635G > T (p.Glu879*), with a frequency of 4/32 (12.5%), which is consistent with previous reports [28]. This variation involves a single nucleotide alteration in exon 10 of *BRCA1*, resulting in an early translation stop signal that leads to nonfunctional protein products. The mutation has been reported in other populations, including Hong Kong [29, 30], Singapore [31], and Malaysia [32]. Additionally, this mutation is also common in ovarian cancer [28]. In the current study, the 4 carriers with the c.2635G > T mutation had HER2-invasive breast cancer, with ages of breast cancer diagnosis recorded at 32, 38, 48, and 53. Three carriers had premenopausal breast cancer, and one had postmenopausal breast cancer. The stage at diagnosis was 1–2, with a high proliferation rate (Ki67 ≥ 30%). Two of the patients had bilateral breast cancer, and one patient had breast cancer with fallopian tube carcinoma, indicating an increased risk of contralateral breast cancer for this mutation. The other two sites with a high frequency of *BRCA1* mutations in this population were c.3756_3759del (p. Ser1253Argfs*10) and c.5072C > A (p. Thr1691Lys). The first variant results in a shift in the reading frame, leading to the loss of *BRCA1* protein function. This variant has been detected in various populations, including South Koreans, Australians, and Poles [33–35]. In the Chinese Hakka population, carriers of this variant presented with invasive breast cancer, HER2-, and higher proliferation (Ki67 ≥ 70%). The age of diagnosis with breast cancer was 28, 48, and 51, with two carriers having premenopausal breast cancer and one having postmenopausal breast cancer. The other variant is the missense variant replaces threonine with lysine at codon 1691 in the BRCT domain of the *BRCA1* protein. Functional studies have reported that this variant impacts *BRCA1* function in transcription activation, protease sensitivity, and peptide binding [36]. This mutation is more common in Chinese individuals [37]. In the Chinese Hakka population, carriers of this variant also presented with invasive breast cancer, HER2-, Ki67 ≥ 60%. The age of breast cancer diagnosis in carriers were 34, 36, and 38, with three carriers developing breast cancer before menopause in this study.

The most frequent mutation in the *BRCA2* gene observed in the Hakka population was c.5164_5165del (p. Ser1722Tyrfs*4), which is consistent with previous reports [28]. This variation results in a shift in the reading frame and loss of function of *BRCA2*. There were seven cases of this mutation in breast cancer patients in the Hakka population. The seven carriers showed high Ki67 expression, with six carriers having HER2- status, and six cases being classified as luminal B breast cancer. The ages at diagnosis with breast cancer for carriers of this mutation were 35, 39, 43, 47, 53, 54, and 58 years, respectively. Four carriers had premenopausal breast cancer, and three carriers had postmenopausal breast cancer. Two carriers had a family history of cancer: one had a family member with rectal cancer, and the other had a family member with ovarian cancer. This variant has been detected in the Chinese Han population [38, 39] and the Macau population [40]. Another high frequency of *BRCA2* mutation in the Hakka population was c.2806_2809del (p. Ala938Profs*21). The ages at diagnosis with breast cancer for carriers of this mutation were 29, 48, and 53, and breast cancer occurred before menopause. The variant has been detected in various populations, including Asians, Europeans, and Americans [41, 42].

The findings from this study revealed that breast cancer patients were diagnosed at a younger age compared to the statistical results of the larger sample of the Hakka population [43]. However, there were no differences observed in the clinical classification, stage, reproductive history, or menstrual status at diagnosis. Compared to non-carriers, the average age at diagnosis of *BRCA1/2* mutation carriers was younger, and more individuals under 40 were diagnosed with breast cancer, which is consistent with the results of previous reports [37, 44, 45]. Multivariate analysis revealed that family members with cancer (OR = 2.36, 95% CI 1.00–5.54, *p* = 0.049) as one of the independent predictors for *BRCA1/2* mutation. Among the thirteen *BRCA1/2* mutation-carrying families, there were nine breast cancer families, two digestive tract cancer families, and two ovarian cancer families. Mutations in the *BRCA* gene might also increase the risk of breast cancer [44, 45], ovarian cancer [46, 47], and colorectal cancer [48, 49]. Bilateral breast cancer has been identified as an independent predictor for *BRCA1/2* mutation. Various studies have shown that the detection rate of *BRCA1/2* gene mutations can be high in patients with bilateral breast cancer [50, 51]. Yuntao Xie et al. recently developed a nomogram, *BRCA*-CRisk, to accurately predict the risk of contralateral breast cancer in patients with *BRCA1/2* mutations [52]. *BRCA1/2* mutation carriers exhibited a higher rate of bilateral breast cancer compared to non-carriers, which is consistent with previous reports [53, 54]. More *BRCA* mutation carriers were HER2- and had no distant metastases (M0 = 88.9%). For individuals carrying *BRCA1* or *BRCA2* mutations and diagnosed with HER2- breast cancer, treatment with PARP inhibitors such as olaparib and talazoparib may be considered [55, 56]. Olaparib has been shown to improve the survival time and quality of life of breast cancer patients, as it is generally well-tolerated with no evidence of cumulative toxicity during extended exposure [7, 57]. Talazoparib is used in patients with advanced breast cancer and a germline *BRCA1/2* mutation [58]. Additionally, Ki67 ≥ 15% was one of the independent predictors for a *BRCA1/2* mutation, as most *BRCA1/2* mutation carriers have breast tumors with vigorous mitosis [27].

The statistics from 2018 indicate that approximately 645,000 (30.9%) cases of premenopausal breast cancer and 1.4 million (69.1%) cases of postmenopausal breast cancer were diagnosed globally. In East Asia, 35.4% of breast cancer cases were premenopausal while 64.6% were postmenopausal [59]. In the Chinese Hakka population, 266 (65.0%) cases of premenopausal breast cancer and 143 (35.0%) postmenopausal breast cancer cases were identified. Among breast cancer *BRCA* mutation carriers, 55 (76.4%) were diagnosed before menopause, which was significantly higher than non-mutation carriers (*P* = 0.026). Univariate logistic regression revealed that premenopausal breast cancer was associated with *BRCA* mutation (OR = 1.93, 95% CI = 1.07–3.48, *P* = 0.028).

The impact of *BRCA1* and *BRCA2* on natural menopausal age remains uncertain and controversial. While some studies have suggested that women with *BRCA1/2* mutations experience an earlier average menopause than women without *BRCA* mutations [60], meta-analyses have not supported this hypothesis [61]. In the study, the average age at natural menopause was 49.60 ± 3.44 years for *BRCA1/2* mutation carriers and 50.52 ± 3.83 years for control subjects, with no significant difference. Breast cancer patients with *BRCA* variants are more likely to occur before menopause. Additionally, there were no significant differences in menstrual duration, menstrual cycle, or fertility between *BRCA1/2* mutation carriers and non-carriers. The analysis of 2295 matched pairs of women with a *BRCA1/2* found that they had similar ages of menarche [62]. Univariate logistic regression showed that age at menarche ≥ 18 years (OR = 1.42, 95% CI = 0.72–2.84, *p* = 0.315) and age at menarche ≤ 13 years (OR = 0.37, 95% CI = 0.13–1.06, *p* = 0.064) were not an independent predictor of *BRCA* mutations. There was no difference in menstrual duration, menstrual cycle [63], or fertility [64] between *BRCA1/2* mutation carriers and non-carriers [65, 66].

*BRCA1/2* genes are important biomarkers for assessing the risk of breast cancer, ovarian cancer, and other related cancers, significantly influencing the choice of individualized treatment for patients. Identifying the hotspot mutation of *BRCA* in China's Hakka population is advantageous for the development of a targeted testing kit focused on specific sites, enabling faster and more cost-effective testing. In addition to identifying hotspot mutations in *BRCA* in the Chinese Hakka population, this study also found that family history, bilateral cancer, HER2-, and Ki67 ≥ 15% are significant independent predictors of *BRCA* pathogenic variants through logistic regression. Based on these clinical features, it is possible to efficiently identify patients who require *BRCA* gene testing. These results provide a basis and reference for clinical consultation and treatment strategies. However, there are some shortcomings in this study. First, this study used the method of inquiry to determine whether the subject was Hakka, and did not analyze the population genetic information of the subjects. Second, this study is based on a single-center retrospective study, and the inclusion of research objects inevitably has selection bias. Third, due to the small number of breast cancer patients carrying *BRCA* gene variants, this study was unable to analyze the differences in clinical characteristics between patients carrying *BRCA1* gene variants and those carrying *BRCA2* gene variants. In the future, a larger sample size of *BRCA* gene mutation research should be carried out in China to find more *BRCA* variants, improve the knowledge of the *BRCA* variation spectrum of Chinese Hakka, and provide a reference for the prevention and treatment of related cancers.

## Conclusions

This article summarizes the *BRCA1/2* mutation sites, clinical and pathological characteristics, menstruation, and fertility status among breast cancer patients in the Chinese Hakka population. The most prevalent pathogenic variant of the *BRCA1* among breast cancer patients was c.2635G > T, while the most common pathogenic variant of the *BRCA2* was c.5164_5165del. A statistical analysis of *BRCA1/2* mutation carriers and non-carriers in Chinese Hakka breast cancer patients revealed that family history, bilateral cancer, HER2-, and Ki67 ≥ 15% were significant independent predictors of *BRCA1/2* pathogenic variants. It is strongly recommended that breast cancer patients with a family history, of bilateral cancer, HER2-, and Ki67 ≥ 15% undergo testing for mutations of the *BRCA1/2* genes. It complemented the *BRCA* gene mutation information in the Chinese population. The findings regarding the relationship between *BRCA* variation and clinicopathological features of breast cancer patients can provide a valuable reference for clinicians in diagnosis and treatment.

### Supplementary Information

**Additional file 1. Supplemental Table 1. **Pathogenic and likely pathogenic *BRCA1/2* variants identified in Chinese Hakka breast cancer patients.

## Data Availability

The variants generated and/or analyzed during the current study will be available in the ClinVar database (https://www.ncbi.nlm.nih.gov/clinvar/). The variants are here [the ClinVar accessions for this data are SCV002520769 to SCV002520941, and SCV003760905 to SCV003760909].

## Abbreviations

FISH: Fluorescence in situ hybridization
TNBC: Triple-Negative Breast Tumors
