# Supplementary material for: BRCA1 and BRCA2 germline mutations in Chinese Hakka breast cancer patients
Source: BMC Med Genomics. 2024 Jan 2;17:3. doi: 10.1186/s12920-023-01772-9 (PMC10763220; doi:10.1186/s12920-023-01772-9)
Supplement: Supplementary file 1 — Additional file 1. Supplemental Table 1. Pathogenic and likely pathogenic BRCA1/2 variants identified in Chinese Hakka breast cancer patients. [file 12920_2023_1772_MOESM1_ESM.docx]

**Supplemental Table1. Pathogenic and likely pathogenic *BRCA1/2* variants identified in Chinese Hakka breast cancer patients**

| Mutation | Exon | Amino acid change | ClinVar | Carrier | Variation type |
| --- | --- | --- | --- | --- | --- |
| *BRCA1* | | | | | |
| c.1A>G | Exon2 | Met1Val | Pathogenic | 1 | Missense |
| c.66dupA | Exon2 | Glu23Argfs*18 | Pathogenic | 1 | Frameshift |
| c.470_471del | Exon7 | Ser157* | Pathogenic | 1 | Nonsense |
| c.938T>G | Exon10 | Leu313* | Pathogenic | 1 | Nonsense |
| c.981_982del | Exon10 | Cys328* | Pathogenic | 1 | Nonsense |
| c.1483G>T | Exon10 | Glu495* | Pathogenic | 1 | Nonsense |
| c.1881_1884del | Exon10 | Ser628Glufs*3 | Pathogenic | 1 | Frameshift |
| c.1960_1961del | Exon10 | Lys654Valfs*18 | Pathogenic | 1 | Frameshift |
| c.1961delA | Exon10 | Lys654Serfs*47 | Pathogenic | 1 | Frameshift |
| c.2253_2254del | Exon10 | Met751Ilefs*10 | Pathogenic | 1 | Frameshift |
| c.2599C>T | Exon10 | Gln867* | Pathogenic | 1 | Nonsense |
| c.2635G>T | Exon10 | Glu879* | Pathogenic | 4 | Nonsense |
| c.2764_2767del | Exon10 | Thr922Leufs*77 | Pathogenic | 1 | Frameshift |
| c.3607C>T | Exon10 | Arg1203* | Pathogenic | 1 | Nonsense |
| c.3756_3759del | Exon10 | Ser1253Argfs*10 | Pathogenic | 3 | Frameshift |
| c.4065_4068del | Exon10 | Asn1355Lysfs*10 | Pathogenic | 1 | Frameshift |
| c.4342delA | Exon12 | Ser1448Alafs*8 | Pathogenic | 1 | Frameshift |
| c.4624_4628del | Exon14 | Ser1542Alafs*30 | Pathogenic | 1 | Frameshift |
| c.4986+5G>A | Intron16 | 4986+5G>A | Likely pathogenic | 1 | Splicing |
| c.5030_5033del | Exon16 | Thr1677Ilefs*2 | Pathogenic | 1 | Frameshift |
| c.5072C>A | Exon16 | Thr1691Lys | Likely pathogenic | 3 | Missense |
| c.5096G>A | Exon17 | Arg1699Gln | Pathogenic | 1 | Missense |
| c.5194-2A>G | Intron19 | 5194-2A>G | Likely pathogenic | 1 | Splicing |
| c.5209dupA | Exon19 | Arg1737Lysfs*93 | Pathogenic | 1 | Frameshift |
| c.5212G>A | Exon19 | Gly1738Arg | Pathogenic | 1 | Missense |
| *BRCA2* | | | | | |
| c.31delT | Exon2 | Phe12Leufs*13 | Pathogenic | 1 | frameshift |
| c.684delT | Exon9 | Asn228Lysfs*2 | Pathogenic | 1 | frameshift |
| c.750_753del | Exon9 | Asp252Valfs*24 | Pathogenic | 1 | frameshift |
| c.1281delC | Exon10 | Leu428Tyrfs*2 | Pathogenic | 1 | frameshift |
| c.1414C>T | Exon10 | Gln472* | Pathogenic | 1 | nonsense |
| c.1528G>T | Exon10 | Glu510* | Pathogenic | 1 | nonsense |
| c.2339C>G | Exon11 | Ser780* | Pathogenic | 2 | nonsense |
| c.2806_2809del | Exon11 | Ala938Profs*21 | Pathogenic | 3 | frameshift |
| c.3109C>T | Exon11 | Gln1037* | Pathogenic | 2 | nonsense |
| c.3715_3716del | Exon11 | Lys1239Thrfs*3 | Pathogenic | 1 | frameshift |
| c.3862_3865del | Exon11 | Lys1289Alafs*3 | Pathogenic | 1 | frameshift |
| c.4525C>T | Exon11 | Gln1509* | Pathogenic | 2 | nonsense |
| c.4790delC | Exon11 | Ser1597Phefs*20 | Pathogenic | 1 | frameshift |
| c.5164_5165del | Exon11 | Ser1722Tyrfs*4 | Pathogenic | 7 | frameshift |
| c.5242delA | Exon11 | Ser1748Alafs*29 | Pathogenic | 1 | frameshift |
| c.5467A>T | Exon11 | Lys1823* | Pathogenic | 1 | nonsense |
| c.5574_5577del | Exon11 | Ile1859Lysfs*3 | Pathogenic | 2 | frameshift |
| c.5682C>G | Exon11 | Tyr1894* | Pathogenic | 1 | nonsense |
| c.5718_5719del | Exon11 | Leu1908Argfs*2 | Pathogenic | 1 | frameshift |
| c.6096dupT | Exon11 | Ile2033Tyrfs*16 | Pathogenic | 2 | frameshift |
| c.6916_6917insA | Exon12 | Ala2306Aspfs*34 | - | 1 | frameshift |
| c.7558C>T | Exon15 | Arg2520* | Pathogenic | 1 | nonsense |
| c.8331+1G>A | Intron 18-19 | - | Pathogenic | 1 | Splicing |
| c.8889dupA | Exon22 | Arg2964Lysfs*54 | - | 1 | frameshift |
| c.8954-5A>G | Intron 22-23 | - | Likely pathogenic | 2 | Splicing |
| c.9400delG | Exon25 | Gly3134Alafs*29 | Pathogenic | 1 | frameshift |
